# Clinical and microbiological efficacy of intra-pocket application of diode laser in grade C periodontitis: a randomized controlled clinical trial

**DOI:** 10.1186/s12903-024-04031-0

**Published:** 2024-02-23

**Authors:** Souzy Kamal Anwar, Amira Mohamed Hafez, Yara Safwat Roshdy

**Affiliations:** 1 Department of Oral Medicine, Periodontology, Oral Diagnosis and Oral Radiology, Faculty of Dentistry, Alexandria University, Champolion St. Azarita, Alexandria, 21521 Egypt; 2 Department of Medical Microbiology and Immunology, Faculty of Medicine, Alexandria University, Champolion St. Azarita, Alexandria, 21521 Egypt

**Keywords:** *Aggregatibacter actinomycetemcomitans*, Antibiotic, Diode laser, Gingival crevicular fluid, Periodontitis, *Porhyromonas Gingivalis*, Real-time polymerase chain reaction

## Abstract

**Background:**

Periodontitis is a microbially induced disease destroying structures anchoring teeth to jaw bones. Although metronidazole in combination with spiramycin is the effective conventional treatment of stage III grade C periodontitis, it has several systemic side effects. Laser therapy is widely used nowadays as an adjunct to scaling and root planing (SRP) to modulate inflammatory host response and eradicate microbes, due to bactericidal and detoxifying effects. Since microbiological analysis is one of the diagnostic methods identifying periodontal risk; our research aimed to investigate the efficacy of intra-pocket application of diode laser (980 nm) versus antibiotic therapy in enhancing clinical and microbiological parameters in stage III grade C periodontitis.

**Methods:**

A randomized controlled clinical trial was conducted on fifty patients with stage III grade C periodontitis, divided equally into two groups. We managed test group by SRP with intra-pocket application of diode laser (980 nm) and the control group by SRP with systemic antibiotic administration (spiramycin and metronidazole). Then, we measured periodontal pocket depth (PPD) and clinical attachment loss (CAL) for both groups, before treatment (baseline), four and twelve weeks after. Moreover, we collected gingival crevicular fluid from both groups at baseline, four and twelve weeks after treatment and analyzed by real-time polymerase chain reaction to detect the relative count of *Aggregatibacter actinomycetemcomitans* and *Porhyromonas gingivalis.*

**Results:**

Compared to baseline, all assessed clinical and microbiological parameters attested improvement at the end of the study period in each group individually with no significant difference between the two studied groups. Although, at twelve weeks, flare up of bacterial levels was detected with systemic antibiotic administration.

**Conclusion:**

Laser therapy can be considered as an effective treatment modality in stage III grade C periodontitis, avoiding the systemic antibiotic side effects and solving the recurrence problems due to bacterial resistance by long term usage.

**Trial registration:**

NCT05222737 retrospectively on 03/02/2022, Clinicaltrial.gov.

## Background

Periodontitis is a microbially induced inflammation of the supporting tissue surrounding teeth that causes irreversible destruction in case causative agents are not removed [1].

The disease’s pathophysiology characterized by key molecular pathways that lead to activation of host-derived proteinases, which propagate marginal periodontal ligament fibers’ loss and apical migration of the junctional epithelium, allowing bacterial biofilm to spread apically along the root surface [2].

Following the new periodontal disease classification (the world workshop 2017), stage III and IV grade C periodontitis is characterized by rapid periodontal attachment loss of 5 mm and bone loss extending to the middle third of the root and beyond, with a rapid rate of progression (radiographic bone loss of 2 mm over 5 years) [3].

Severe periodontitis (grade C) formerly called aggressive periodontitis is caused by an imbalance of a variety of microorganisms, host response, and modifying factors [4]. The microbial biofilm is an important protagonist in the etiology and progression of periodontitis [4]. Given the importance of biofilm, periodontal therapy should aim to prevent inflammatory responses by reducing and eliminating these microbial active factors [5]. Stage III grade C periodontitis is primarily caused by the bacteria *Aggregatibacter actinomycetemcomitans* (*Aa*), whereas stage IV grade C periodontitis is caused by *Porphyromonas gingivalis* (*Pg*), *Tannerella forsythia*, and *Aa*, Gram-negative coccobacillus, capnophile, and microaerophilic bacteria [4].

Appropriate diagnosis can identify the severity, activity and progression of periodontitis, which is important for reaching the best treatment plan. Nowadays, the novel methods of diagnosis can identify periodontal risk and predict its progression and thus allow earlier detection of the disease. Those methods include microbiological analysis and biomarkers in oral fluid [6]. Real-time PCR has been demonstrated to be a sensitive and rapid method for detecting and quantifying individual microbial species [7]. The majority of real-time PCR tests rely on detecting bacterial small-subunit 16 S rRNA sequences [8]. All bacterial species have multiple copies of this DNA subunit, which contains highly conserved species-specific sequences [8].

Conventional treatment of stage III grade C periodontitis with metronidazole in combination with spiramycin in addition to SRP, is an effective treatment of active periodontitis eliminating most of the pathogenic bacteria [9]. However, systemic antimicrobials cause several side effects, the most important of which is the development of resistant species, despite controlling or eradicating many pathogens [10].

Consequently, the bactericidal and detoxifying effects of lasers have been proposed as an alternative treatment modality to facilitate non-surgical periodontal treatment providing good results using 980 nm diode laser [11]. Additional use of the diode laser with stage III grade C periodontitis has shown to be more effective than SRP alone in terms of clinical, biochemical and microbiological parameters [5]. The diode laser energy improves healing by interacting strongly with inflamed tissues, removing the biofilm within the necrotic tissue of pocket wall [12].

To our knowledge, comparisons between conventional treatment by antibiotics and diode laser have been rarely investigated. Therefore, we were interested to further investigate the efficacy of intra-pocket application of diode laser versus systemic antibiotic therapy in management of stage III grade C periodontitis using clinical and microbiological analytical parameters.

The null hypothesis was that there would be no significant difference in clinical and microbiological parameters following treatment of stage III grade C periodontitis patients with diode laser in comparison with systemic antibiotic.

## Methods

### Study design

We conducted a two-arm parallel randomized controlled clinical trial on fifty patients diagnosed with severe periodontitis (stage III grade C). Patients were recruited from the outpatient clinic of the Department of Oral Medicine, Periodontology, Diagnosis and Oral Radiology, Faculty of Dentistry, Alexandria University, Egypt.

### Ethical approval

The study was accepted by the Research Ethics Committee of the Faculty of Dentistry, Alexandria University, Egypt (IRB NO: 00010556-IORG0008839). It was also performed in accordance with principles of the modified Helsinki code for human clinical studies (2013) [13] and CONSORT guidelines (2010) for reporting randomized clinical trials [14]. The protocol of this study was explained to all subjects and/or their legal guardian(s) and their informed consent was obtained. Registration of the study was done at U.S. National Institutes of Health Clinical Trials Registry (NCT05222737).

### Inclusion and exclusion criteria

We included patients that were systemically healthy, of both sexes, with an age between 15 and 35 years old having severe periodontitis (stage III grade C) with interdental clinical attachment loss (CAL) ≥ 5, probing depth (PD) ≥ 6 mm and rapid rate of bone loss showed by panoramic radiograph [1]. Eligibility criteria also included patients with no history of previously taken any antibiotic therapy for the past three months [5]. We excluded patients if they had history of smoking, usage of antimicrobial mouthwash during the previous 3 weeks, with < 20 teeth present and pregnant or lactating women [5].

### Sample size estimation

Sample size was estimated assuming 5% alpha error and study power of 80% [15]. Reported mean difference log^10^ colony forming units (CFU) *P.gingivalis* after 6 months when antibiotics were used = 4.1, confidence interval = 1.9, 6.3, and calculated standard deviation (SD) = 2.63 [16]. Reported mean ± SD log10 CFU *P.gingivalis* at baseline = 9.22 ± 1.63 and at 6 months after laser therapy = 7.00 ± 1.10, making the mean difference = 2.22. Based on comparison of means, the minimum sample size was calculated, using G*power 3.1.9.4 sample size calculator, to be 20 per group which was increased to 25 to control for attrition (withdrawal) bias. The total sample size required = number of groups × number per group = 2 × 25 = 50 [17, 18].

### Grouping and randomization of the study participants

Fifty participants diagnosed with severe periodontitis (stage III grade C) were randomly assigned into two equal groups: Group I (test) was managed by SRP with intra-pocket application of diode laser (980 nm) and Group II (control) was managed by SRP with systemic antibiotic administration (spiramycin and metronidazole).

Simple randomization of subjects fulfilled the eligibility criteria was carried out using a computer-generated list of random numbers (https://www.sealedenvelope.com/simple-randomiser/v1/lists). The list was generated using random allocation software. Each allocation was represented by a code and sealed in sequentially numbered opaque envelopes that were opened at the time of the intervention.

### Intervention

After completion of baseline measurements for both clinical parameters and microbiological evaluation, the two groups received full mouth SRP using hand instruments and ultrasonic scalers and oral hygiene instructions.

**Fig. 1:**
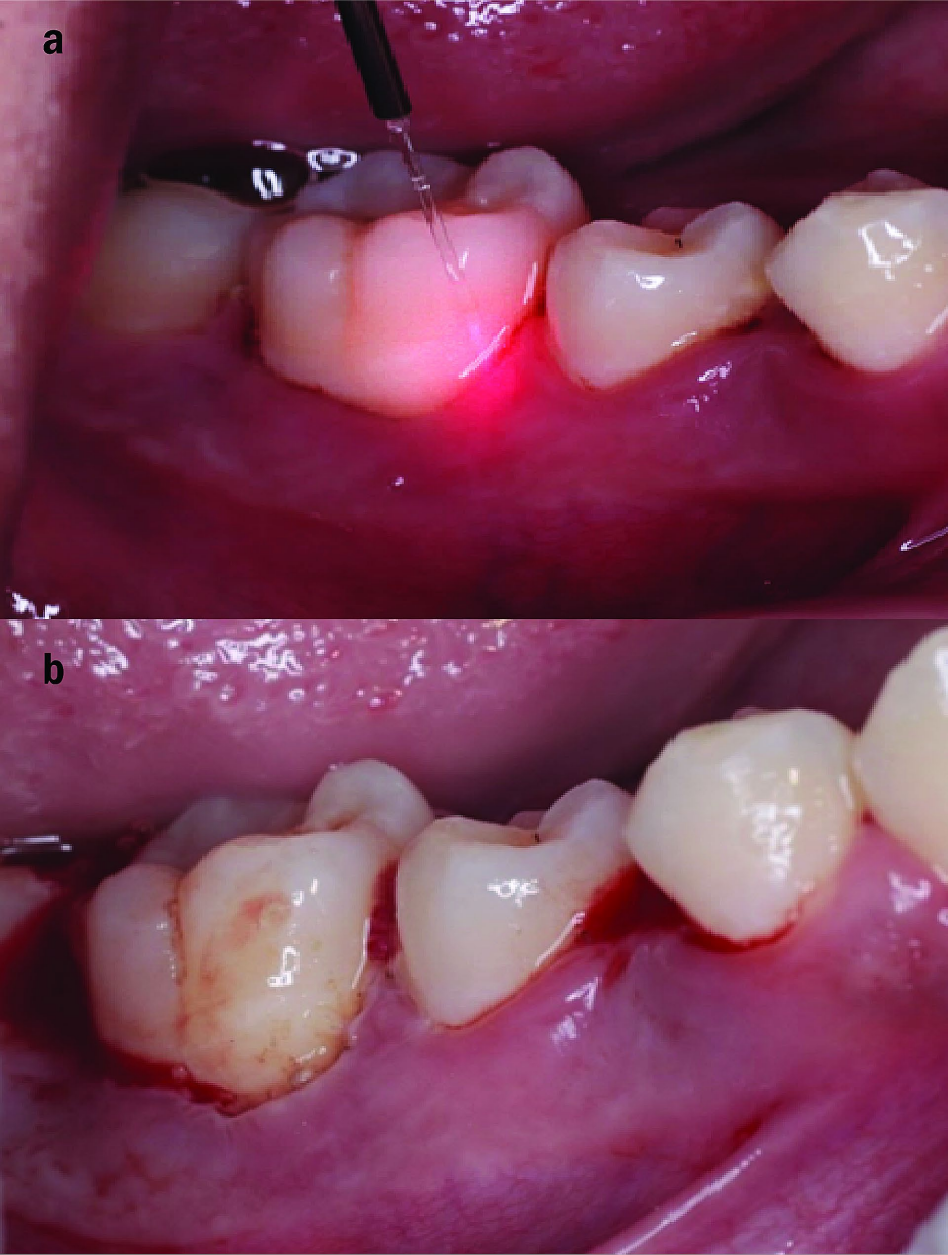
Clinical photographs showing **a**) Intra pocket diode laser application in test group. **b**) Fresh bleeding after laser application in test group

The test group underwent a treatment using a 980-nm diode laser (Medency Primo, Piazza della Libertà, 49, 36,077 Altavilla Vicentina VI, Italy) in continuous contact focused mode at 2 watts power, with a flexible glass fiberoptic of 300-µm spot diameter and power density of 2830 W/cm^2^. The total energy per unit area (fluency) was 94.3 J/cm^2^ [5, 12]. Our previously stated protocol [19] was followed, which involved calibrating and inserting the laser fiberoptic to be 1 mm less than the measured pocket depth. This shortening by 1 mm allowed absorption of laser energy around its tip and irradiation of the pathogenic periodontal tissues without thermal damage to healthy tissues. The laser was then activated, and the tip was initiated after the fiber had reached the calibrated depth. The fiber was then inserted in light contact parallel to the root surface towards the diseased soft tissue lining of the pocket. Then it was moved with a sweeping motion at a constant speed of 2.5 mm/sec, starting from the base of the pocket, and moving upward maintaining contact with the soft tissue lining of the pocket. The procedure was repeated until the entire circumference of the root was irradiated, and fresh bleeding was observed. The total irradiation period was approximately 30 s per pocket. This allowed laser-assisted soft-tissue curettage. After fulfilling laser irradiation, we rinsed with thorough saline solution to prevent heat injury to the root surface [5, 20]. Between treatments of each tooth, the fiber tip was cleaned with damp gauze followed by 5 s of wiping with ethanol to prevent the buildup of debris. The end of the tip was cut and tested prior to and between successive treatments to ensure good beam emission [5]. (Fig. 1a,b).

For control group, the patients were given a combination of Spiramycin 1.5 minimum inhibitory concentration and Metronidazole 250 mg (Spirazole forte®, pharoina pharmaceuticals, Helioplis - Cairo, Egypt) at a dosage of 17 mg per Kg, taken on an empty stomach for two weeks. We discontinued the antibiotic for a period of eight weeks and then re-administered three times daily for the following two weeks [21].

### Clinical parameters assessment

Periodontal clinical parameters including: PPD and CAL were recorded at baseline, four and twelve weeks after treatment. All measurements were recorded at tested sites using a periodontal probe (UNC-15; Hu-Friedy, Chicago, IL), before getting gingival crevicular fluid (GCF) samples, by a blinded pre-calibrated clinicians [22, 23]. Calibration was performed for two examiners prior to the study, inter- and intra-examiner reliability were calculated; and kappa ranged from 0.82 to 0.88 indicating excellent agreement between examiners and across time [24].

### Microbiological sampling technique

**Fig. 2:**
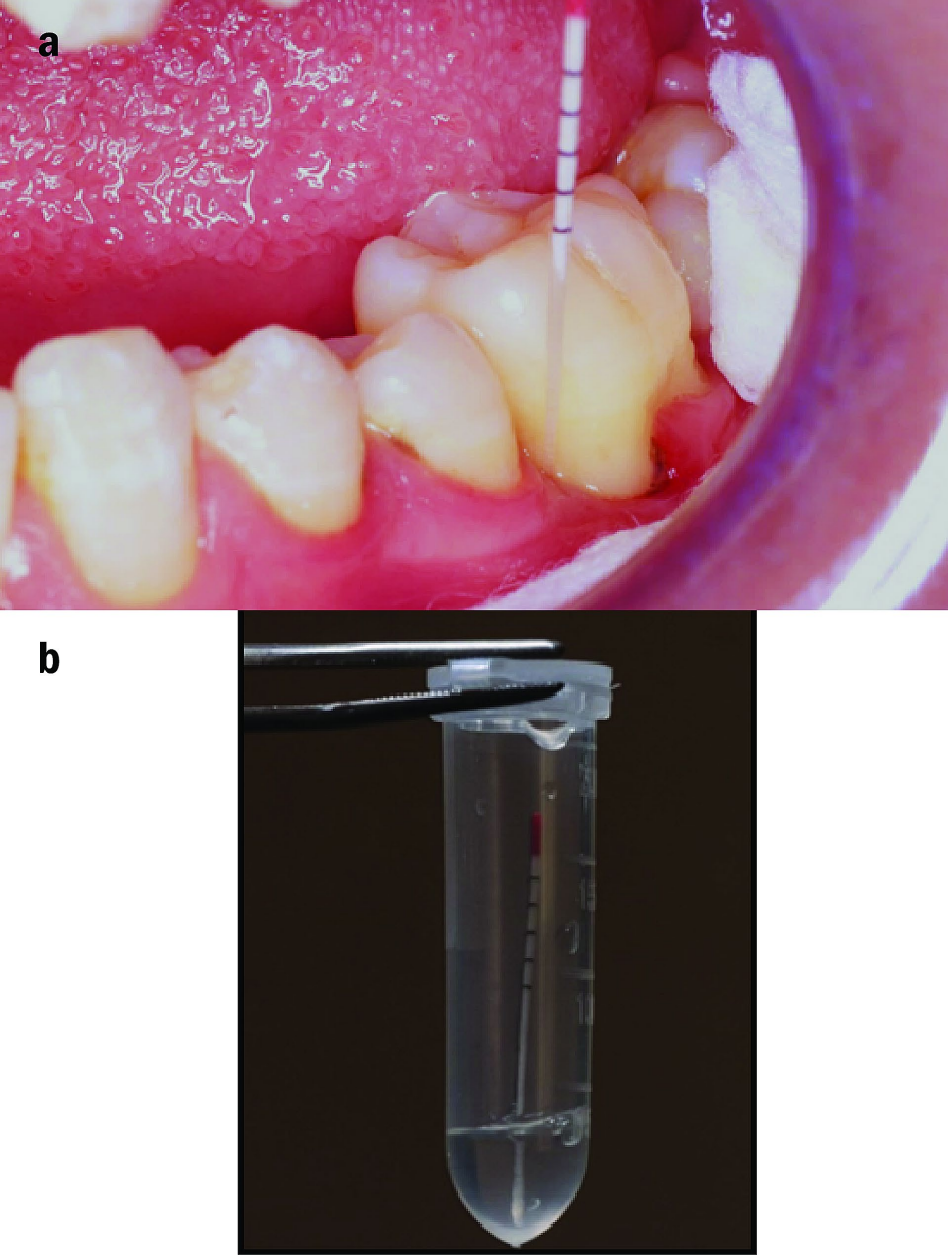
Clinical photographs showing **a**) GCF collection by paper point. **b**) Eppendorf tube containing 500 µl phosphate-buffered saline and the collected GCF sample

In all patients, we collect GCF samples from the area showing the deepest pocket depth to analyze the two bacteria (*Aa* and *Pg*), at baseline, four- and twelve-weeks following treatment. To permit specimen analysis, all sites to be sampled were isolated using cotton rolls and gently air dried. For each site GCF was collected using sterile paper points in intra pocket depth until minimal resistance was felt. Then we transported the paper points an Eppendorf tube containing 200 ml sterile saline to Alexandria Main University Hospital Microbiology laboratory. Any paper points contaminated with blood was discarded. Privacy & confidentiality of the samples and the information from the samples were assured. We used Code to label the samples. We stored the tubes at -20 °C till we performed DNA extraction and quantitation of the bacteria by real-time polymerase chain reaction (PCR) method [25, 26] (Fig. 2a,b).

### SYBR Green Real-Time PCR assays for P.gingivalis and A. actinomycetemcomitans

Oligonucleotide primers (as shown in Table 1) were targeting the 16 S rRNA gene (rDNA) sequences of the bacteria (universal primer set, recognizing domain bacteria), *Pg* and *Aa*. Amplification was performed in a light cycler (Rotor Gene Q, Qiagen, Germany). Forward and reverse primers (4 pmol each) were used in 20 µl total reactions volume containing 10 µl 2x Maxima SYBR Green qPCR kit, and 2 µl of the DNA extract. PCR amplification was performed with initial denaturation at 95 ˚C for 10 min, followed by 40 cycles of denaturation at 95 ˚C for 30 s, annealing at 60 ˚C for 1 min, and extension at 72 ˚C for 30 s. Melting curve analysis was performed from 40 to 95 ˚C with a plate reading step after every 1 ˚C and held at a particular temperature for 10 s to check the specificity of the product formed. Quantitation of the targeted bacterial DNA was expressed as relative quantitation and calculated automatically by the Rotor-Gene. The cycle threshold (Ct) at which DNA of a specific target was detected relative to the Ct at which universal bacterial DNA was detected [27, 28].

**Table 1:** Oligonucleotide primers targeted the 16 S rRNA gene (rDNA) sequences of the bacteria

Bacteria	Primer Name	Primer Sequence (5’-3’)	Product bp
***Total bacteria***	UnivF	5′-CGCTAGTAATCGTGGATCAGAATG-	69
	UnivR	5′-TGTGACGGGCGGTGTGTA-3′	
***Aa***	F	5′- GCGAACGTTACGCGTTTTAC-3′	220
	R	5′- GGCAAATAAACGTGGGTGAC-3′	
***Pg***	F	*5′-TGGTTTCATGCAGCTTCTTT-3′*	*126*
	R	*5′-TCGGCACCTTCGTAATTCTT-3′*	

### Statistical analysis

Data was fed to the computer and analyzed using IBM SPSS software package version 20.0. (Armonk, NY: IBM Corp). The Shapiro-Wilk test was used to verify the normality of distribution. Comparisons between groups for categorical variables was assessed using Chi-square test (Monte Carlo). The Mann Whitney test was used to compare two groups for not normally distributed quantitative variables. Friedman test was used for not normally distributed quantitative variables, to compare between more than two periods and Post Hoc Test (Dunn’s) for pairwise comparisons. The significance of the obtained results was judged at the 5% level.

## Result

Fifty individuals with age ranging from 17 to 35 years, consisting of 22 males and 28 females, who met the eligibility criteria were included in this study after screening 65 potentially eligible patients. They underwent baseline evaluations and were randomly assigned to study groups, as shown in **Table 2**. Each group consist of twenty-five patients suffering from stage III grade C periodontitis (CAL > 5). The three-month clinical trial was successfully completed by all participants. None of the patients stated any oral health problems during the study.

**Table 2:** Comparison between the two studied groups according to demographic data

	Test (*n* = 25)		Control (*n* = 25)		Test of Sig.	*p*
	No.	%	No.	%		
**Sex**						
Male	14	56.0	8	32.0	χ^2^= 2.922	0.087
Female	11	44.0	17	68.0		
**Age (years)**						
Min. – Max.	19.0–34.0		17.0–35.0		t = 1.762	0.084
Mean ± SD.	28.52 ± 5.64		25.40 ± 6.82			
Median (IQR)	30.0(21.0–33.0)		27.0(23.0–29.0)			

**Fig. 3:**
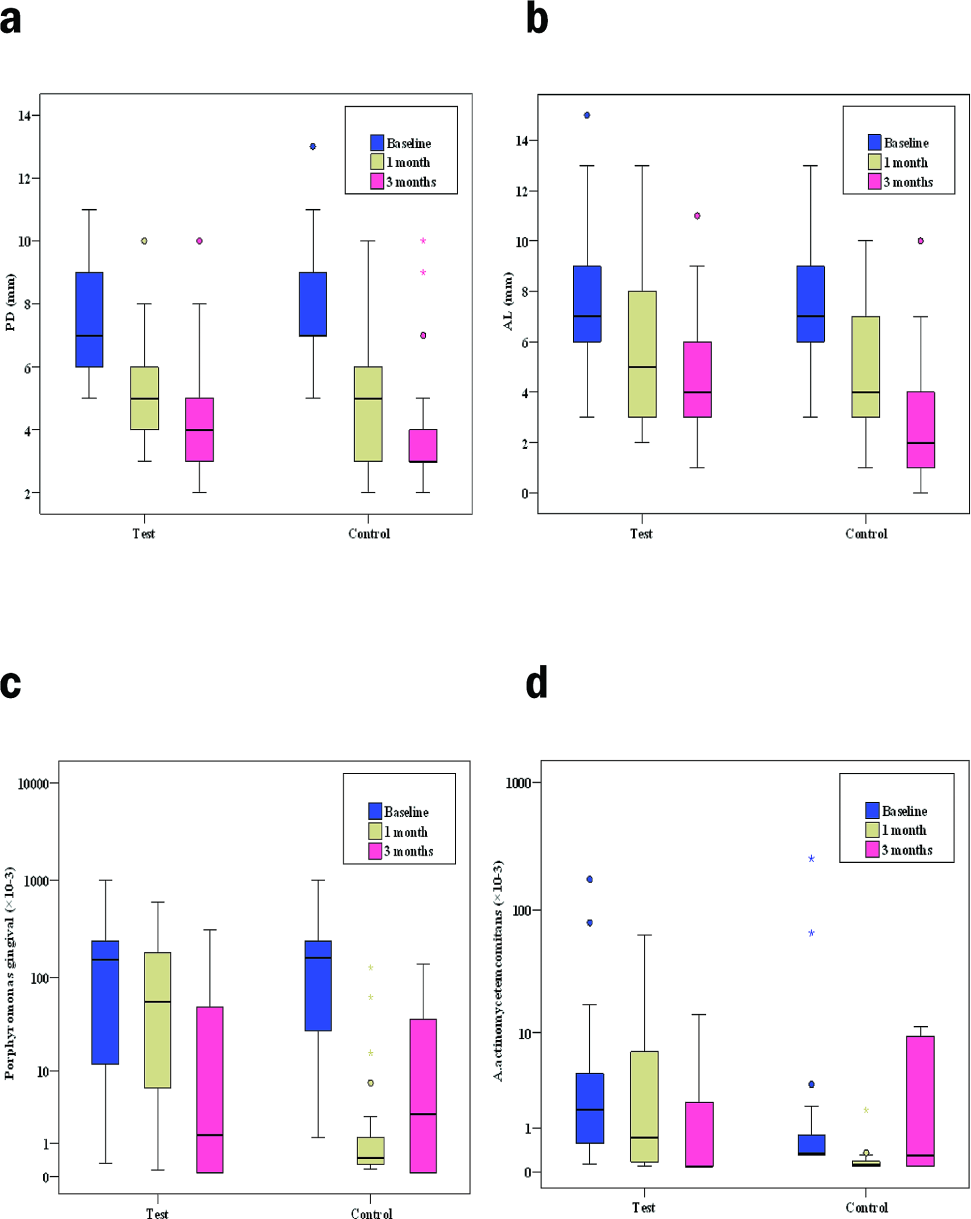
Representative graphs showing **a**) Comparison between the three studied periods according to periodontal probing pocket depth (PD) in each group. **b**) Comparison between the three studied periods according to attachment loss (AL) in each group. **c**) Comparison between the three studied periods according to porphyromonas gingivalis in each group. **d**) Comparison between the three studied periods according to A.actinomycetemcomitans in each group

Concerning PPD, at baseline there was no statistically significance difference between the two groups allowing for un biased comparison, also there was no statistically significant difference between the two groups at the three times points (*p* = 0.0423, 0.219, 0.089 respectively). However, there was a highly statistically significant decrease in PPD comparing between the standard periods (*p* < 0.001^*^) in each of the test and control group (Table 3; Fig. 3a).

Although, the significant decrease in the PPD in control group from baseline to 3 months (median from 7.0 to 3.0) was greater than that in test group (median from 7.0 to 4.0) there was no statistically significance difference between the two groups at the end of the treatment (*p* = 0.089) (Table 3; Fig. 3a).

Concerning CAL, on comparing both groups, they showed non-statistically significant difference at three times points (*P* = 1.000, 0.125, 0.078 respectively). (Table 3 and Graph 2)

However, there was a highly statistically significant decrease in CAL comparing the studied periods in each of the test and control groups (*p* < 0.001^*^) (Table 3; Fig. 3b).

On the other hand, the significant decrease in the CAL in control group from baseline to 3 months (median from 7.0 to 2.0) was greater than that in test group (median from 7.0 to 4.0) with no statistically significance difference between the two groups at the end of the treatment (*p* = 0.078) (Table 3; Fig. 3b).

Regarding *Pg* level intracreviculary, there was high statistically significance decrease in both groups from baseline to the end of the treatment yet, there was no statistically significant difference between test and control groups at baseline and 3 months respectively (*p* = 0.793,0701) (Table 3; Fig. 3c).

A noticeable point, there was a highly statistically significant difference between two groups on 1 month (*p* < 0.001^*^), where the control group showed an early rapid decrease in the level of *Pg* (median from 160.0 to 0.41) in comparison to test group (median from 150.0 to 55.1). However, on the 3 months the control group showed a slight increase in the level of *Pg* (median from 0.41 to 2.95) in comparison to the steady decrease in the test group (median from 55.1 to 1.39) (Table 3; Fig. 3c).

Moreover, on comparing between the two groups, there was a highly statistically significant decrease from 1 month to 3 months interval in the test group (*p* = 0.001^*^) with no statistically significant difference in control group (*p* = 0.572) (Table 3; Fig. 3c).

As for intracrevicular level of *Aa*, there was no statistically significant difference between both test and control groups at baseline and 3 months (*p* = 0.056, 0.187 respectively) (Table 3; Fig. 3d).

On the other hand, there was a highly statistically significant difference between two groups on 1 month (*p* = 0.001^*^), where the control group showed an early rapid decrease in the level of *Aa* (median from 0.25 to 0.03) in comparison to test group (median from 1.78 to 0.66) (Table 3; Fig. 3d).

However, on the 3 months the control group showed an increase in the level of *Aa* (median from 0.03 to 0.22) in comparison to the steady decrease in the test group (median from 0.66 to 0.0) (Table 3; Fig. 3d).

Furthermore, on comparing each group in the first month and third month, there was a highly statistically significant decrease in the test group (*p* = 0.048^*^) while statistically significant increase in control group (*p* = 0.007^*^) (Table 3; Fig. 3d).

In both groups, from baseline to 3 months, the clinical parameters concerning PPD and CAL has significantly been improved in correlation with the significant decrease in the microbiological changes concerning the relative count or the levels of *Pg* and *Aa* (Table 3).

**Table 3:** Comparison between the three studied periods according to different parameters in each group

		Baseline	1 month	3 months	Fr (p_0_)	Pairwise
**PPD (mm)**	**Test (** *n* ** = 25)**					
	Mean ± SD.	7.56 ± 1.85	5.52 ± 1.83	4.52 ± 1.96	41.892^*^ (< 0.001^*^)	p_1_ < 0.001^*^,p_2_ < 0.001^*^,p_3_ = 0.007^*^
	Median (Min. – Max.)	7 (5–11)	5 (3–10)	4 (2–10)		
	**Control (** *n* ** = 25)**					
	Mean ± SD.	8.0 ± 1.91	4.92 ± 2.20	3.88 ± 2.05	45.516^*^ (< 0.001^*^)	p_1_ < 0.001^*^,p_2_ < 0.001^*^,p_3_ = 0.048^*^
	Median (Min. – Max.)	7 (5–13)	5 (2–10)	3 (2–10)		
	**U (p)**	272.00 (0.423)	250.00 (0.219)	227.00 (0.089)		
**CAL (mm)**	**Test (** *n* ** = 25)**					
	Mean ± SD.	7.56 ± 2.84	5.68 ± 2.95	4.84 ± 3.05	41.231^*^ (< 0.001^*^)	p_1_ < 0.001^*^,p_2_ < 0.001^*^,p_3_ = 0.016^*^
	Median (Min. – Max.)	7.0 (3.0–15.0)	5.0 (2.0–13.0)	4.0 (1.0–11.0)		
	**Control (** *n* ** = 25)**					
	Mean ± SD.	7.40 ± 2.60	4.44 ± 2.74	3.48 ± 3.04	41.692^*^ (< 0.001^*^)	p_1_ < 0.001^*^,p_2_ < 0.001^*^,p_3_ = 0.028^*^
	Median (Min. – Max.)	7.0 (3.0–13.0)	4.0 (1.0–10.0)	2.0 (0.0–10.0)		
	**U (p)**	312.50 (1.000)	234.0 (0.125)	222.50 (0.078)		
**Pg (×10** ^**− 3**^ **)**	**Test (** *n* ** = 25)**					
	Mean ± SD.	186.7 ± 224.3	141.0 ± 187.0	54.49 ± 93.63	15.360^*^ (< 0.001^*^)	p_1_ = 1.000,p_2_ = 0.001^*^ ,p_3_ = 0.001^*^
	Median (Min. – Max.)	150(0.23–1000)	55.1(0.05–0.593)	1.39(0–309)		
	**Control (** *n* ** = 25)**					
	Mean ± SD.	188.5 ± 215.7	8.9 ± 27.24	23.96 ± 39.15	24.320^*^ (< 0.001^*^)	p_1_ < 0.001^*^,p_2_ < 0.001^*^ ,p_3_ = 0.572
	Median (Min. – Max.)	160(1.27–1000)	0.41(0.09–125)	2.95(0.0–36.3)		
	**U (p)**	299.00 (0.793)	94.0^*^ (< 0.001^*^)	293.00 (0.701)		
**Aa (×10 −** ^**3**^**)**	**Test (** *n* ** = 25)**					
	Mean ± SD.	12.71 ± 37.09	6.32 ± 13.46	2.05 ± 3.95	16.333^*^ (< 0.001^*^)	p_1_ = 0.048^*^,p_2_ < 0.001^*^,p_3_ = 0.048^*^
	Median (Min. – Max.)	1.78 (0.04–174.0)	0.66 (0.0–63.20)	0.0 (0.0–14.20)		
	**Control (** *n* ** = 25)**					
	Mean ± SD.	13.29 ± 51.65	0.13 ± 0.35	4.08 ± 4.76	19.567^*^ (< 0.001^*^)	p_1_ < 0.001^*^,p_2_ = 0.104,p_3_ = 0.007^*^
	Median (Min. – Max.)	0.25(0.22–253.2)	0.03 (0.0–1.75)	0.22 (0.0–11.30)		
	**U (p)**	214.0 (0.056)	141.0^*^ (0.001^*^)	247.0 (0.187)		

SD: **Standard deviation U: Mann Whitney test** χ^2^: **Chi square test** MC: **Monte Carlo**

**Fr**: **Friedman test**, Sig. bet. periods was done using **Post Hoc Test** (**Dunn’s)**

p: p value for comparing between **Test** and **Control** in each period

p_0_: p value for comparing between the three studied periods in each group

p_1_: p value for comparing between **Baseline** and **1 month**

p_2_: p value for comparing between **Baseline** and **3 months**

p_3_: p value for comparing between **1 month** and **3 months**

*: Statistically significant at *p* ≤ 0.05

## Discussion

Gingival inflammation is initiated by bacterial biofilm formation; however, periodontitis initiation and progression are dependent on symbiotic ecological changes in the microbiome in response to nutrients come from gingival inflammatory and tissue breakdown products, as well as anti-bacterial mechanisms that tries to contain the microbial challenge once inflammation has begun in the gingival sulcus. This activates several key molecular pathways, which in turn activate host-derived proteinases, allowing for the loss of marginal periodontal ligament fibers, junctional epithelial apical migration, and bacterial biofilm apical spread along the root surface [3]. As a result, the primary characteristics of periodontitis are the loss of periodontal tissue support, manifested as clinical attachment loss and radiographic alveolar bone loss, the presence of periodontal pocketing, and gingival bleeding [1].

Hence, periodontal therapy aims to reduce or remove pathogenic bacteria besides limiting and preventing the inflammatory disease process [1, 29]. Clinicians face a challenge in treating aggressive periodontitis since there are no established guidelines or protocols for effective disease management. Scaling and root planing is the first conventional method of non-surgical treatment for the management of stage III grade C; periodontitis formerly known as aggressive periodontitis [30]. However, it was demonstrated that due to the periodontal bacteria’ capacity to infiltrate periodontal tissues, SRP does not entirely eradicate them from deep periodontal tissues [31, 32].

Antibiotics have proved to produce positive results, when combined with SRP [33]. Research has demonstrated that spiramycin and metronidazole are an efficient therapy for active periodontitis [33, 34]. However, in addition to their systemic side effects, it has been confirmed that utilizing antibiotics results in bacterial resistance [10, 34, 35].

Laser periodontal therapy has been advocated as an adjunctive or alternative for traditional mechanical periodontal treatments since 1994 [36]. It has been demonstrated that several advantageous characteristics of lasers, such as their hemostatic effects, calculus removal, or bactericidal activity and detoxification against periodontal bacteria, improve the treatment outcomes [37].

Numerous types of lasers have been applied to periodontics. These include semiconductor diode lasers that range in wavelength from 610 nm (red) to 980 nm (infrared) and Nd:YAG lasers emitting light at 1064 nm. Both pulsed wave (PW) and continuous wave (CW) modes can be used with it. Depending on how it interacts with the tissue surface, the laser light may be transmitted, absorbed, dispersed, or refracted. The wavelength, power, and optical characteristics of the tissue determine whether the laser energy absorbed by the tissues causes thermal, coagulation, or vaporization effects [38]. Due to its strong absorption by blood hemoglobin, diode laser light is a great option for removing the highly vascularized, inflammatory tissues found in the periodontal pocket [37, 39]. In addition, periodontal pathogenic microorganisms are eliminated by the thermal and photo-disruptive effects of a diode laser (wavelength 980 nm, 2 watts power) [40]. Laser light absorption raises tissue temperatures, which makes the majority of nonsporulating bacteria, including anaerobes, easily inactivated [41, 42]. When utilized for subgingival curettage and periodontal pocket disinfection, soft tissue lasers, such as these semiconductor diodes and the Nd:YAG lasers emitting light at 1064 nm, have variable degrees of effectiveness [37, 43]. However, as it has been shown that lasers are unable to remove calcified deposits from the root surface, they should only be used as a supplement to mechanical periodontal therapy [44].

Thus, the effectiveness of laser therapy compared to conventional antibiotic therapy on PPD, CAL parameters, and GCF levels of *Aa* and *Pg* in stage III grade C periodontitis piqued our curiosity.

To the best of our knowledge, this is the first study that compared intra-pocket diode laser therapy (980 w) for one session with systemic antibiotic (combination of spiramycin and metronidazole) as adjunctive with SRP in stage III grade C periodontitis investigating the gingival crevicular levels of *Aa* and *Pg* in addition to clinical evaluation based on PPD, CAL.

The current study found a statistically significant decrease in PPD and CAL in the laser group between the baseline and three months after therapy. This was consistent with several studies that employed various diode laser wavelengths ranging from 660 to 980 nm and photodynamic therapy (PDT) with varied photosensitizers and follow-up times to arrive at the same conclusion as our study [5, 12, 45–49]. While there was a substantial decrease in PPD in the 3-month follow-up period, Annaji et al. [45] found no significant difference in CAL when comparing the diode laser group with PDT laser group. Additionally, Talamac et al. [50] and Ertugrul et al. [51], employing a different kind of laser (Er,Cr:YSGG laser), concurred with our results. In contrast to SRP-only, they discovered that the SRP plus Er,Cr:YSGG laser treatment in aggressive periodontitis was more effective in improving the clinical periodontal parameters (PD + CAL).They explained this in light of research showing that Er,Cr:YSGG enhance cell attachment and migration on the root surfaces.

Other researches asserted the effect of laser irradiation which enhance connective tissue attachment through de-epithelization of periodontal pockets and promotion of wound healing by favorably influencing the cells needed for this process, such as fibroblasts and endothelial cells, as well as by increasing collagen synthesis [46, 52, 53].

In other trials, antibiotics combination as metronidazole-amoxicillin, spiramycin-metronidazole, ciprofloxacin-metronidazole, metronidazole alone, doxycycline and metronidazole-amoxicillin–clavulanic acid when used with SRP, they reduced PPD and CAL significantly compared to SRP alone giving a beneficial outcome over 3,6 or 12 months follow up and remained stable for 1 year [54–56].

In line with Quee et al.‘s [57] findings, who used systemic rodogyl (spiramycin and metronidazole) three tablets twice daily for 14 days and observed a greater improvement in CAL and PPD at intervals of two to six months in advanced periodontal disease, the PPD and CAL results for the antibiotic group in our study showed a statistically significant difference from baseline to three months after therapy. A different antibiotic (clarithromycin) was also employed by Andere et al. [58], who also found that the antibiotic and antibiotic plus PDT groups had considerably lower probing depths than the other groups. The clinical attachment level did, however, improve statistically only in the antibiotic plus PDT group.

A greater amount of healing of the tissues in the pocket’s apical region was achieved as a result of the pathogenic bacteria found subgingivally being more successfully eliminated by the combination of spiramycin and metronidazole due to their synergistic effect against a variety of infections as well as a wider spectrum of activity [57].

In our study comparing the two groups, the improvement of PPD and CAL in antibiotic group from baseline to 3 months was greater than that in laser group with no statistically significance difference between them. On the contrary, another found that SRP plus antibiotic (amoxicillin + metronidazole 7 days, 3 times daily) significantly reduced CAL and PPD compared to SRP plus PDT using diode laser (810 nm) [59]. By eradicating subgingival microorganisms that are still present after SRP, metronidazole’s positive effect seeks to boost the host defense system in infection. The secret to the effectiveness of systemic antibiotics in treating periodontal disorders may lie in how sensitive bacteria are to them [59]. However, in agreement with our findings, others reported that in stage III and IV grade C periodontitis treated with PDT for four sessions, clinical parameters were significantly reduced [60].

New diagnostic tests for periodontitis, according to most studies, cannot replace the conventional procedures, although they can be used in conjunction. Traditional diagnostic approaches depict signs connected to pathological processes associated with periodontal disease, as bleeding on probing, deep probing depths, and clinical attachment loss [6, 61]. These approaches offer a variety of advantages, including cost-effectiveness and convenience of use, as well as clinically valuable information about the illness site and the presence or absence of damaged tissues. However, they have a lot of flaws, such as failing to offer relevant information on periodontal inflammatory morbidity or outcome [62].

Microbiological diagnosis, is one of the most recent approaches for diagnosing periodontitis which is useful in bacterial detection and quantification either from saliva or gingival crevicular fluid [63].

For periodontitis, qualitative testing are bacterial diagnostic methods that identify the presence but not the quantity of microorganisms. Because periodontal disease is caused by endogenous bacteria, periodontal bacteria can be found in both healthy gingival sulcus and diseased periodontal pockets, making qualitative approaches ineffective for diagnosing periodontal disease. Analysis of changes in bacterial counts after and before periodontal treatment, which enables us to assess the effectiveness of periodontal treatment, is the most crucial application of microbiological investigation in periodontal disease. For this, quantitative bacteria examinations are required [63–65].^(48–50)^

Real-time PCR has lately grown in popularity for quantitative detection of periodontal microorganisms [64, 66]. This approach, which was first used to count the copies of DNA, has been used to quantify the number of bacteria [65].

The levels of *Pg* and *Aa* in each group individually decreased from baseline to 3 months following therapy, according to the current study, in a statistically significant way. Although the levels of 2 bacteria reduced more in the laser group than in the antibiotic group at the end of treatment, there was no statistically significant difference between the two groups.

The test group’s *Pg* and *Aa* levels decreased steadily from the baseline to the end of the treatment. This is consistent with prior studies, despite using varied diode laser wavelengths [5, 12, 45]. Additionally, other investigations using PDT with a diode laser at a wavelength of 670 nm for a single or series of sessions with a three-month follow-up reached the same conclusions [45, 48, 49, 67]. This proves that periodontal lesions are a key cause of inflammation and bone resorption and supports the laser’s bactericidal and detoxifying effects by reducing bacterial load and inflammation via lowering the levels of prostaglandin E2, which increase in periodontal connective tissues [12, 68–70].

According to many publications, the most effective treatment option for deep periodontal pockets that keeps the levels of all bacterial species at extremely low levels even 12 weeks following therapy is intra pocket diode laser therapy (980 nm) plus SRP [12, 20, 53]. The best bacterial reduction was recorded two weeks after therapy and then significantly reduced after 12 weeks. They attributed this result to laser irradiation’s capacity to eradicate pathogenic bacteria from dentinal tubules, which serve as a reservoir for recolonization and pocket infection [71, 72].

Derdilopoulou et al. [73] used Er:YAG Laser and sonic instruments in contrast to our study. However, these methods failed to reduce *Aa* in periodontal pockets. Bacterial reduction achieved in his study did not retain three months after therapy.

According to Chan et al. [74], wavelength and energy density are crucial factors, for laser treatment. Low-power lasers that have the appropriate wavelength, dosage, along with the right photosensitizer can have a viable bactericidal effect. They concluded that providing 60 s of irradiation with a diode laser of appropriate power 100 mW and wavelength 830 nm could be a useful supplement to mechanical debridement in preventing pathogenic germs from colonizing subgingival lesions.

Concerning the *Pg* and *Aa* levels in our study’s antibiotic group. There was a rapid early decrease in the levels of both bacteria from baseline to 1 month then the levels slightly increased from 1 month to the end of the study. This may be explained by the bacterial resistance of antibiotic in accordance with Amano et al. [75] who explained that the bacteria decreased rapidly by 1 month by the effect of antibiotic administration for 2 weeks then some of the bacteria resist the effect of second administration of antibiotic so its level increased slightly from 1 month to the end of the study.

In line with our findings, Johnson et al. [76] used different systemic antibiotic (amoxicillin and metronidazole) found that total bacteria including *Aa* reduced significantly from baseline to 3 months, with the exception of *Pg* as it was able to recolonize in subgingival sites in which it had been suppressed shortly after active periodontal treatment. Additionally, Ardila et al. [77] demonstrated that *Pg* and *Aa* isolates were resistant to amoxicillin, azithromycin, and metronidazole. This is because *Pg* and *Aa* have a variety of strategies for evading the host immune system along with their high virulence, which enables them to affect human tissues and cause periodontal disease. These microorganisms have also developed sophisticated ways for developing antibiotic resistance [75].

The decline in levels of *Pg* and *Aa* following laser therapy is a predictor of the therapeutic effect and the state of the patient’s condition. Nonetheless, the current study has some limitations. These include the length of the follow-up period. Therefore, further studies with longer follow up period are still needed and would allow more solid conclusions to be drawn. Furthermore, studies are also needed to assess the treatment outcomes using different types of lasers with different setting parameters using photosensitizer and multiple treatment sessions versus local delivery antibiotic. Moreover, additional studies test the efficacy of combing intra-pocket laser therapy with 3-day systemic antibiotic, evading antibiotic resistance with long term usage. Additionally, research supported by histological and clinical analysis is also needed to be conducted to give confirming evidence using latest analysis regarding the advantageous effect of laser therapy on the levels of *Pg* and *Aa* in the management of severe periodontitis (stage III grade C).

## Conclusion

Within the limitation of this study, we can conclude that both treatment modalities can be used in management of stage III grade C periodontitis. However, due to the flare-up of bacterial levels brought on by bacterial resistance that develops after systemic antibiotic re-administration, the combination of SRP and intra-pocket diode laser therapy has better outcomes. This is proven by its superior ability to reduce the levels of *Pg* and *Aa* in GCF when compared to systemic antibiotic administration. Therefore, intra-pocket diode laser therapy can be considered an effective treatment modality for stage III grade C periodontitis evading the antibiotic resistance problems and side effects.

## Data Availability

All data included in this current study are available from the corresponding author upon request.

## Abbreviations

SRP: Scaling and root planing
PPD: Probing pocket depth
CAL: Clinical attachment loss:*Aa*:*Aggregatibacter actinomycetemcomitans*
*Pg*: *Porphyromonas gingivalis*
PD: Probing depth
CFU: Colony forming units
GCF: Gingival crevicular fluid
PCR: Polymerase chain reaction
PDT: Photodynamic therapy

## References

[1] Tonetti MS, Greenwell H, Kornman KS (2018). Staging and grading of periodontitis: Framework and proposal of a new classification and case definition. J Clin Periodontol.

[2] Muñoz-Carrillo JL, Hernández-Reyes VE, García-Huerta OE, Chávez-Ruvalcaba F, Chávez-Ruvalcaba MI, Chávez-Ruvalcaba KM. Díaz-Alfaro L: Pathogenesis of Periodontal; 2020.

[3] Papapanou PN, Sanz M, Buduneli N, Dietrich T, Feres M, Fine DH, Flemmig TF, Garcia R, Giannobile WV, Graziani F (2018). Periodontitis: Consensus report of workgroup 2 of the 2017 World workshop on the classification of Periodontal and Peri-implant diseases and conditions. J Periodontol.

[4] Könönen E, Müller HP (2014). Microbiology of aggressive periodontitis. Periodontol 2000.

[5] Matarese G, Ramaglia L, Cicciù M, Cordasco G, Isola G (2017). The effects of Diode Laser Therapy as an Adjunct to Scaling and Root Planing in the treatment of aggressive periodontitis: a 1-Year randomized controlled clinical trial. Photomed Laser Surg.

[6] Kinney JS, Morelli T, Oh M, Braun TM, Ramseier CA, Sugai JV, Giannobile WV (2014). Crevicular fluid biomarkers and periodontal disease progression. J Clin Periodontol.

[7] Boutaga K, van Winkelhoff AJ, Vandenbroucke-Grauls CM, Savelkoul PH (2003). Comparison of real-time PCR and culture for detection of Porphyromonas gingivalis in subgingival plaque samples. J Clin Microbiol.

[8] Heid CA, Stevens J, Livak KJ, Williams PM (1996). Real time quantitative PCR. Genome Res.

[9] Poulet PP, Duffaut D, Barthet P, Brumpt I (2005). Concentrations and in vivo antibacterial activity of spiramycin and metronidazole in patients with periodontitis treated with high-dose metronidazole and the spiramycin/metronidazole combination. J Antimicrob Chemother.

[10] Vergidis PI, Falagas ME (2008). Multidrug-resistant Gram-negative bacterial infections: the emerging threat and potential novel treatment options. Curr Opin Investig Drugs.

[11] Borrajo JL, Varela LG, Castro GL, Rodríguez-Nuñez I, Torreira MG (2004). Diode laser (980 nm) as adjunct to scaling and root planing. Photomed Laser Surg.

[12] Kamma JJ, Vasdekis VG, Romanos GE (2009). The effect of diode laser (980 nm) treatment on aggressive periodontitis: evaluation of microbial and clinical parameters. Photomed Laser Surg.

[13] World Medical Association (2013). Declaration of Helsinki: ethical principles for medical research involving human subjects. JAMA.

[14] Moher D, Hopewell S, Schulz KF, Montori V, Gøtzsche PC, Devereaux PJ, Elbourne D, Egger M, Altman DG (2010). CONSORT 2010 explanation and elaboration: updated guidelines for reporting parallel group randomised trials. BMJ.

[15] Guerrero A, Nibali L, Lambertenghi R, Ready D, Suvan J, Griffiths GS, Wilson M, Tonetti MS (2014). Impact of baseline microbiological status on clinical outcomes in generalized aggressive periodontitis patients treated with or without adjunctive Amoxicillin and metronidazole: an exploratory analysis from a randomized controlled clinical trial. J Clin Periodontol.

[16] Euzebio Alves VT, de Andrade AK, Toaliar JM, Conde MC, Zezell DM, Cai S, Pannuti CM, De Micheli G (2013). Clinical and microbiological evaluation of high intensity diode laser adjutant to non-surgical periodontal treatment: a 6-month clinical trial. Clin Oral Investig.

[17] Faul F, Erdfelder E, Lang AG, Buchner A (2007). G*Power 3: a flexible statistical power analysis program for the social, behavioral, and biomedical sciences. Behav Res Methods.

[18] Petrie A, Sabin C. Medical statistics at a glance. Wiley; 2019.

[19] Hafez AM, Mohamed FR, Anwar SK, THE EFFECT OF INTRA-POCKET APPLICATION OF DIODE LASER ON THE MOBILITY OF TEETH WITH GRADE C PERIODONTITIS (2023). Alexandria Dent J.

[20] Manjunath S, Singla D, Singh R (2020). Clinical and microbiological evaluation of the synergistic effects of diode laser with nonsurgical periodontal therapy: a randomized clinical trial. J Indian Soc Periodontol.

[21] Zambon JJ, Christersson LA, Genco RJ (1986). Diagnosis and treatment of localized juvenile periodontitis. J Am Dent Assoc.

[22] Lenox JA, Kopczyk RA (1973). A clinical system for scoring a patient’s oral hygiene performance. J Am Dent Assoc.

[23] Glavind L, Löe H (1967). Errors in the clinical assessment of periodontal destruction. J Periodontal Res.

[24] McHugh ML (2012). Interrater reliability: the kappa statistic. Biochem Med (Zagreb).

[25] Ashimoto A, Chen C, Bakker I, Slots J (1996). Polymerase chain reaction detection of 8 putative periodontal pathogens in subgingival plaque of gingivitis and advanced periodontitis lesions. Oral Microbiol Immunol.

[26] Guentsch A, Kramesberger M, Sroka A, Pfister W, Potempa J, Eick S (2011). Comparison of gingival crevicular fluid sampling methods in patients with severe chronic periodontitis. J Periodontol.

[27] Suzuki N, Yoshida A, Saito T, Kawada M, Nakano Y (2004). Quantitative microbiological study of subgingival plaque by real-time PCR shows correlation between levels of Tannerella forsythensis and Fusobacterium Spp. J Clin Microbiol.

[28] Yoshida A, Suzuki N, Nakano Y, Kawada M, Oho T, Koga T (2003). Development of a 5’ nuclease-based real-time PCR assay for quantitative detection of cariogenic dental pathogens Streptococcus mutans and Streptococcus sobrinus. J Clin Microbiol.

[29] Teles RP, Haffajee AD, Socransky SS (2006). Microbiological goals of periodontal therapy. Periodontol 2000.

[30] Teughels W, Dhondt R, Dekeyser C, Quirynen M (2014). Treatment of aggressive periodontitis. Periodontol 2000.

[31] Lafaurie GI, Mayorga-Fayad I, Torres MF, Castillo DM, Aya MR, Barón A, Hurtado PA (2007). Periodontopathic microorganisms in peripheric blood after scaling and root planing. J Clin Periodontol.

[32] Mombelli A, Schmid B, Rutar A, Lang NP (2000). Persistence patterns of Porphyromonas gingivalis, Prevotella intermedia/nigrescens, and Actinobacillus actinomyetemcomitans after mechanical therapy of periodontal disease. J Periodontol.

[33] Loesche WJ (1999). The antimicrobial treatment of periodontal disease: changing the treatment paradigm. Crit Rev Oral Biol Med.

[34] Rotzetter PA, Le Liboux A, Pichard E, Cimasoni G (1994). Kinetics of spiramycin/metronidazole (Rodogyl) in human gingival crevicular fluid, saliva and blood. J Clin Periodontol.

[35] Quee TC, Roussou T, Chan EC (1983). In vitro activity of rodogyl against putative periodontopathic bacteria. Antimicrob Agents Chemother.

[36] Aoki A, Ando Y, Watanabe H, Ishikawa I (1994). In vitro studies on laser scaling of subgingival calculus with an erbium:YAG laser. J Periodontol.

[37] Aoki A, Sasaki KM, Watanabe H, Ishikawa I (2004). Lasers in nonsurgical periodontal therapy. Periodontol 2000.

[38] Cobb CM (2006). Lasers in periodontics: a review of the literature. J Periodontol.

[39] Cobb CM, Low SB, Coluzzi DJ (2010). Lasers and the treatment of chronic periodontitis. Dent Clin North Am.

[40] Moritz A, Schoop U, Goharkhay K, Schauer P, Doertbudak O, Wernisch J, Sperr W (1998). Treatment of periodontal pockets with a diode laser. Lasers Surg Med.

[41] Coluzzi DJ (2008). Fundamentals of lasers in dentistry: basic science, tissue interaction, and instrumentation. J Laser Dent.

[42] Gutknecht N. Proceedings of the 1st international workshop of evidence based dentistry on lasers in dentistry: Quintessence Publishing (IL); 2007.

[43] Centty IG, Blank LW, Levy BA, Romberg E, Barnes DM (1997). Carbon dioxide laser for de-epithelialization of periodontal flaps. J Periodontol.

[44] Tucker D, Cobb CM, Rapley JW, Killoy WJ (1996). Morphologic changes following in vitro CO2 laser treatment of calculus-ladened root surfaces. Lasers Surg Med.

[45] Annaji S, Sarkar I, Rajan P, Pai J, Malagi S, Bharmappa R, Kamath V (2016). Efficacy of photodynamic therapy and lasers as an Adjunct to Scaling and Root Planing in the treatment of aggressive periodontitis - A clinical and microbiologic short term study. J Clin Diagn Res.

[46] Saafan A, El-Nahass H, Nasr AS, Radwan R (2013). Effect of low power diode laser 810 nm on TGF-β1 level in GCF in aggressive periodontitis. J Dent Lasers.

[47] de Oliveira RR, Schwartz-Filho HO, Novaes AB, Taba M (2007). Antimicrobial photodynamic therapy in the non-surgical treatment of aggressive periodontitis: a preliminary randomized controlled clinical study. J Periodontol.

[48] Chitsazi MT, Shirmohammadi A, Pourabbas R, Abolfazli N, Farhoudi I, Daghigh Azar B, Farhadi F (2014). Clinical and Microbiological effects of Photodynamic Therapy Associated with non-surgical treatment in aggressive periodontitis. J Dent Res Dent Clin Dent Prospects.

[49] Borekci T, Meseli SE, Noyan U, Kuru BE, Kuru L (2019). Efficacy of adjunctive photodynamic therapy in the treatment of generalized aggressive periodontitis: a randomized controlled clinical trial. Lasers Surg Med.

[50] Talmac AC, Yayli NZA, Calisir M, Ertugrul AS (2022). Comparing the efficiency of Er,Cr:YSGG laser and diode laser for the treatment of generalized aggressive periodontitis. Ir J Med Sci.

[51] Ertugrul AS, Tekin Y, Talmac AC (2017). Comparing the efficiency of Er,Cr:YSGG laser and diode laser on human β-defensin-1 and IL-1β levels during the treatment of generalized aggressive periodontitis and chronic periodontitis. J Cosmet Laser Ther.

[52] Kreisler M, Al Haj H, d’Hoedt B (2005). Clinical efficacy of semiconductor laser application as an adjunct to conventional scaling and root planing. Lasers Surg Med.

[53] Qadri T, Javed F, Johannsen G, Gustafsson A (2015). Role of diode lasers (800–980 nm) as adjuncts to scaling and root planing in the treatment of chronic periodontitis: a systematic review. Photomed Laser Surg.

[54] Sanz M, Herrera D, Kebschull M, Chapple I, Jepsen S, Beglundh T, Sculean A, Tonetti MS (2020). Treatment of stage I-III periodontitis-the EFP S3 level clinical practice guideline. J Clin Periodontol.

[55] Theodoro LH, Lopes AB, Nuernberg MAA, Cláudio MM, Miessi DMJ, Alves MLF, Duque C, Mombelli A, Garcia VG (2017). Comparison of repeated applications of aPDT with Amoxicillin and metronidazole in the treatment of chronic periodontitis: a short-term study. J Photochem Photobiol B.

[56] Rabelo CC, Feres M, Gonçalves C, Figueiredo LC, Faveri M, Tu YK, Chambrone L (2015). Systemic antibiotics in the treatment of aggressive periodontitis. A systematic review and a bayesian network meta-analysis. J Clin Periodontol.

[57] Quee TC, Chan EC, Clark C, Lautar-Lemay C, Bergeron MJ, Bourgouin J, Stamm J (1987). The role of adjunctive rodogyl therapy in the treatment of advanced periodontal disease. A longitudinal clinical and microbiologic study. J Periodontol.

[58] Bechara Andere NMR, Dos Santos NCC, Araujo CF, Mathias IF, Rossato A, de Marco AC, Santamaria M, Jardini MAN, Santamaria MP (2018). Evaluation of the local effect of nonsurgical periodontal treatment with and without systemic antibiotic and photodynamic therapy in generalized aggressive periodontitis. A randomized clinical trial. Photodiagnosis Photodyn Ther.

[59] Arweiler NB, Pietruska M, Pietruski J, Skurska A, Dolińska E, Heumann C, Auschill TM, Sculean A (2014). Six-month results following treatment of aggressive periodontitis with antimicrobial photodynamic therapy or Amoxicillin and metronidazole. Clin Oral Investig.

[60] Al-Khureif AA, Mohamed BA, Siddiqui AZ, Khan AA, Divakar DD (2020). Repeated application of photodynamic and antibiotic therapy as an adjunct to root surface debridement in patients with grade C and stage III or IV aggressive periodontitis. Photodiagnosis Photodyn Ther.

[61] Buduneli N. Biomarkers for periodontal diseases. Biomarkers in Periodontal Health and Disease: Rationale, benefits, and future directions. edn.: Springer; 2020. pp. 41–58.

[62] Armitage GC (1995). Clinical evaluation of periodontal diseases. Periodontol 2000.

[63] Yoshida A, Ansai T. Microbiological diagnosis for periodontal diseases. Periodontal diseases–A Clinician’s Guide 2012:55–66.

[64] Suzuki N, Nakano Y, Yoshida A, Yamashita Y, Kiyoura Y (2004). Real-time TaqMan PCR for quantifying oral bacteria during biofilm formation. J Clin Microbiol.

[65] Yoshida A, Suzuki N, Nakano Y, Oho T, Kawada M, Koga T (2003). Development of a 5’ fluorogenic nuclease-based real-time PCR assay for quantitative detection of Actinobacillus actinomycetemcomitans and Porphyromonas gingivalis. J Clin Microbiol.

[66] Suzuki N, Yoshida A, Nakano Y (2005). Quantitative analysis of multi-species oral biofilms by TaqMan Real-Time PCR. Clin Med Res.

[67] Moreira AL, Novaes AB, Grisi MF, Taba M, Souza SL, Palioto DB, de Oliveira PG, Casati MZ, Casarin RC, Messora MR (2015). Antimicrobial photodynamic therapy as an adjunct to non-surgical treatment of aggressive periodontitis: a split-mouth randomized controlled trial. J Periodontol.

[68] Novaes AB, Schwartz-Filho HO, de Oliveira RR, Feres M, Sato S, Figueiredo LC (2012). Antimicrobial photodynamic therapy in the non-surgical treatment of aggressive periodontitis: microbiological profile. Lasers Med Sci.

[69] Caruso U, Nastri L, Piccolomini R, d’Ercole S, Mazza C, Guida L (2008). Use of diode laser 980 nm as adjunctive therapy in the treatment of chronic periodontitis. A randomized controlled clinical trial. New Microbiol.

[70] Salvi GE, Beck JD, Offenbacher S (1998). PGE2, IL-1 beta, and TNF-alpha responses in diabetics as modifiers of periodontal disease expression. Ann Periodontol.

[71] Giuliana G, Ammatuna P, Pizzo G, Capone F, D’Angelo M (1997). Occurrence of invading bacteria in radicular dentin of periodontally diseased teeth: microbiological findings. J Clin Periodontol.

[72] Gutknecht N, Franzen R, Schippers M, Lampert F (2004). Bactericidal effect of a 980-nm diode laser in the root canal wall dentin of bovine teeth. J Clin Laser Med Surg.

[73] Derdilopoulou FV, Nonhoff J, Neumann K, Kielbassa AM (2007). Microbiological findings after periodontal therapy using curettes, Er:YAG laser, Sonic, and ultrasonic scalers. J Clin Periodontol.

[74] Chan Y, Lai CH (2003). Bactericidal effects of different laser wavelengths on periodontopathic germs in photodynamic therapy. Lasers Med Sci.

[75] Amano A, Chen C, Honma K, Li C, Settem RP, Sharma A (2014). Genetic characteristics and pathogenic mechanisms of periodontal pathogens. Adv Dent Res.

[76] Johnson JD, Chen R, Lenton PA, Zhang G, Hinrichs JE, Rudney JD (2008). Persistence of extracrevicular bacterial reservoirs after treatment of aggressive periodontitis. J Periodontol.

[77] Ardila CM, Bedoya-García JA (2020). Antimicrobial resistance of Aggregatibacter actinomycetemcomitans, Porphyromonas gingivalis and Tannerella forsythia in periodontitis patients. J Glob Antimicrob Resist.

